# Supplementary material for: Lung and Intercostal Upper Abdomen Ultrasonography for Staging Patients with Ovarian Cancer: A Method Description and Feasibility Study
Source: Diagnostics (Basel). 2020 Feb 5;10(2):85. doi: 10.3390/diagnostics10020085 (PMC7167950; doi:10.3390/diagnostics10020085)
Supplement: Supplementary file 1 [file diagnostics-10-00085-s001.pdf]

**Supplementary Materials:** The following are available online at www.mdpi.com/xxx/s1,

Video S1: Lung ultrasonograms of the left lower pleural space and upper abdomen. Nodules on the left diaphragmatic pleural and abdominal surfaces, pleural effusions, ascites and normal spleen.

Video S2: Lung ultrasonograms and corresponding surgical video of the right pleural space and upper abdomen. Bulky tumors on the right diaphragmatic pleural and abdominal surfaces and pleural effusions.

Video S3: Lung and intercostal upper abdomen ultrasonograms and corresponding computed tomography of the right pleural space and upper abdomen. Solid-cystic tumor between the liver and the diaphragm, close to the mid-line.

Video S4: Intercostal upper abdomen ultrasonograms and corresponding surgical video of the left and right upper abdomen. Omental infiltration protruding on the posterior spleen surface, plaque lesion on the right posterior abdominal surface of the diaphragm; significant and small amounts of pleural effusions in the right and left pleural spaces respectively.

Video S5: Intercostal upper abdomen ultrasonograms and corresponding surgical video of the right upper abdomen. Infiltration of the right abdominal surface of the diaphragm.

Video S6: Intercostal upper abdomen ultrasonograms and corresponding surgical video of the right upper abdomen. Involvement of the ligamentum teres of the liver and the hepatic hilum.

Video S7: Intercostal upper abdomen ultrasonograms and corresponding surgical video of the left upper abdomen. Nodular lesion in the spleen hilum.

Video S8: Lung ultrasonograms and corresponding computed tomography of the right pleural space. Pleural effusions, lung consolidation and inflammation, metastatic parenchymal lung lesion and diaphragm thickening.

Video S9: Intercostal upper abdomen ultrasonograms and images from surgery of the right upper abdomen. Small amount of pleural effusions; a "bright line" of the normal diaphragm appearance (no lesions) in ultrasound; and miliary diaphragm peritoneum carcinomatosis in surgical image.
